# Molecular Characterization of Mutant Mouse Strains Generated from the EUCOMM/KOMP-CSD ES Cell Resource

**DOI:** 10.1007/s00335-013-9467-x

**Published:** 2013-08-04

**Authors:** Edward Ryder, Diane Gleeson, Debarati Sethi, Sapna Vyas, Evelina Miklejewska, Priya Dalvi, Bishoy Habib, Ross Cook, Matthew Hardy, Kalpesh Jhaveri, Joanna Bottomley, Hannah Wardle-Jones, James N. Bussell, Richard Houghton, Jennifer Salisbury, William C. Skarnes, Sanger Mouse Genetics Project, Ramiro Ramirez-Solis

**Affiliations:** The Wellcome Trust Sanger Institute, Hinxton, Cambridgeshire, CB10 1SA UK

## Abstract

The Sanger Mouse Genetics Project generates knockout mice strains using the EUCOMM/KOMP-CSD embryonic stem (ES) cell collection and characterizes the consequences of the mutations using a high-throughput primary phenotyping screen. Upon achieving germline transmission, new strains are subject to a panel of quality control (QC) PCR- and qPCR-based assays to confirm the correct targeting, cassette structure, and the presence of the 3′ *LoxP* site (required for the potential conditionality of the allele). We report that over 86 % of the 731 strains studied showed the correct targeting and cassette structure, of which 97 % retained the 3′ *LoxP* site. We discuss the characteristics of the lines that failed QC and postulate that the majority of these may be due to mixed ES cell populations which were not detectable with the original screening techniques employed when creating the ES cell resource.

**Electronic supplementary material:**

The online version of this article (doi:10.1007/s00335-013-9467-x) contains supplementary material, which is available to authorized users.

## Introduction

**Fig. 1:**
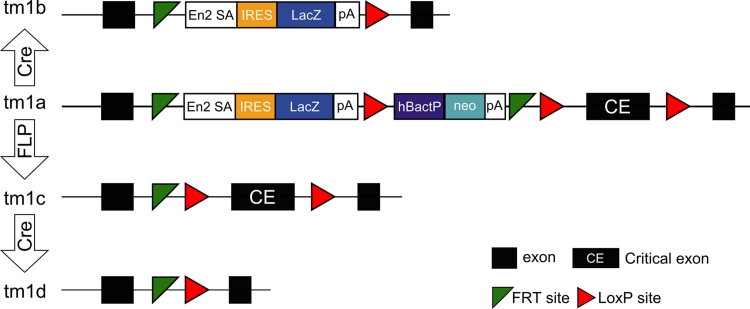
EUCOMM/KOMP-CSD allele structure and conversion. The EUCOMM/KOMP-CSD allele “knockout-first” allele (tm1a) contains an IRES:*lacZ* trapping cassette and a floxed promoter-driven *neo* cassette inserted into the intron of the targeted gene. The presence of an Engrailed (En2) splice acceptor disrupts gene function, resulting in a *lacZ* fusion for studying gene expression localisation. Exposure to a source of *Flp* recombinase removes the gene trap cassette, converts the “knockout-first” allele to a conditional allele (tm1c) and restores the gene’s activity. Subsequent exposure to *Cre* recombinase will then delete the floxed exon of the tm1c allele resulting in a frameshift and null mutation (tm1d). *Cre* recombinase can also be used to convert the tm1a allele to the tm1b form and generate a nonconditional *lacZ*-tagged null allele without the promoter-driven *neo* cassette

The extensive genetic resources available for the mouse, including the sequencing and annotation of the genomes of multiple inbred laboratory strains (Church et al. 2009; Keane et al. 2011; Flicek et al. 2012; Wong et al. 2012), have facilitated comprehensive comparisons with the human genome (Guigo et al. 2003; Zheng-Bradley et al. 2010; Mouse ENCODE Consortium et al. 2012). This makes the mouse a powerful tool for both investigating gene function and modelling disease progression in mammalian systems. This importance can be demonstrated by the wealth of resources available for researchers studying human diseases and genetic disorders, including (but not limited to) cancer (Frese and Tuveson 2007; Kim and Baek 2010; Leystra et al. 2012), visual (Gao et al. 2002; van de Pavert et al. 2007) and auditory dysfunctions (Leibovici et al. 2008; Spiden et al. 2008), neurodegenerative conditions (Games et al. 1995; Schilling 1999; Ravikumar et al. 2004; Wirths and Bayer 2010), and diabetes (Cho et al. 2001; Duan et al. 2004). There are over 1,100 human diseases with one or more mouse models, and over 3,600 mouse genotypes model human disease as reported at the Mouse Genome Database (MGD) (http://www.informatics.jax.org, December 2012). To facilitate these investigations, several large-scale efforts to create knockout mutations in mice have been established (Bradley et al. 2012) by the systematic construction of targeted mutations (Valenzuela et al. 2003; Prosser et al. 2011; Skarnes et al. 2011). Currently, the largest resource of targeted mutations is the EUCOMM/KOMP-CSD mouse embryonic stem cell (ESC) collection (Skarnes et al. 2011), which is based on JM8 agouti or non-agouti C57BL/6N ES cells (Pettitt et al. 2009). The structure and modification of the promoter-driven “knockout-first” EUCOMM/KOMP-CSD allele, which forms the majority of the collection, is shown in Fig. 1.

The EUCOMM/KOMP-CSD collection, along with those generated by Regeneron, and the Canadian NorComm programme form the International Mouse Knockout Consortium (IKMC) resource (Collins et al. 2007; Ringwald et al. 2011; Bradley et al. 2012) and are the main source of ES cells used for mouse production by the International Mouse Phenotyping Consortium (IMPC) (Brown and Moore 2012).

The goal of the IMPC is to generate knockout strains for all protein-coding genes in the mouse on a pure C57BL/6N genetic background, and to elucidate gene function by use of a broad-spectrum high-throughput primary phenotyping screen. These phenotypes can then be studied in more depth by the scientific community at large within specialized areas of interest.

The aims of the IMPC overlap with the Wellcome Trust Sanger Mouse Genetics Project (Sanger MGP) (White et al. 2013) which was formed in 2006 to generate and phenotype 200 mutant mouse strains per year using a battery of tests designed to detect changes in a variety of systems, including metabolism, dysmorphology, behaviour, cardiovascular, immunity, visual and auditory response, viability, and homozygous lethality (Ayadi et al. 2012). Strains are available to the scientific community directly from Sanger Institute while colonies are actively breeding, and from the European Mutant Mouse Archive (Wilkinson et al. 2010) or KOMP Repository (Lloyd 2011) once archived. The primary phenotypic data are also readily available at the Sanger Mouse Portal (http://www.sanger.ac.uk/mouseportal).

At the time of writing, the EUCOMM/KOMP-CSD ES clone collection consisted of targeted clones for 12,350 genes, 56 % of the 22,147 CCDS (Pruitt et al. 2009) gene models present in Ensembl (Flicek et al. 2012). The resource was generated by use of a high-throughput modular gateway-based vector construction and positive–negative selection for high-efficiency targeting in ES cells (Skarnes et al. 2011). Clones were then screened by long-range PCR and sequencing to confirm targeting and the presence of the 3′ *loxP* site that is required for the conditionality of the mutant allele. Although this approach is appropriate for a high-throughput pipeline in terms of cost and speed, it does have its limitations. For example, long-range PCR is likely to miss mutations within the cassette and is not able to detect mixed ESC populations. As the resource is exploited to generate mouse lines, it will be important to ascertain the molecular structure of the alleles transmitted to mice.

Here we present a detailed and extensive molecular characterization of the mutant alleles in mouse strains generated from the resource. We demonstrate that although the majority of the mouse lines produced by Sanger MGP from the EUCOMM/KOMP-CSD collection are correct, some problematic events were detected. We have developed a set of quality control (QC) criteria and assays to screen out affected strains as early as possible following germline transmission of the incorrect alleles.

## Materials and Methods

The care and use of all mice in this study were in accordance with the UK Home Office regulations, UK Animals (Scientific Procedures) Act of 1986, and were approved by the Wellcome Trust Sanger Institute Ethical Review Committee.

### A Minimum Standard for Mouse QC

The high-throughput nature of the Sanger MGP makes it impractical to apply a QC strategy based on Southern blot analysis. Thus, our QC strategy is centred on a set of PCR-based methods configured to detect abnormalities in the identity of the targeted gene, the presence/absence of the 3′ *loxP* site, and the number of vector insertions. Our strategy complies with the IKMC guidelines to include at least one test in each of the four different categories (Table 1).

**Table 1:** IKMC minimum allele QC standards

QC category	QC test (at least one per category)	Stage
Confirm targeting of the allele	Southern blot with *neo* or external probe	ESC or mice
	Loss of wild-type allele (LoA) qPCR	Mice
	5′ and 3′ LRPCR	Mice
	Absence of a WT-specific short-range PCR (srPCR) product in homozygous mice	Mice
	Gene expression analysis on mRNA or protein	Mice
Confirm structure of the cassette	srPCR on various parts of the cassette (e.g., mutant-specific srPCR, *lacZ*, *neo*, cassette ends, *neo*, or *lacZ* count by qPCR	Mice
Confirm conditionality of the tm1a allele	Gene-specific or universal srPCR to detect the *loxP* site 3′ to the CE	Mice
Confirm absence of additional insertions	Southern blot with *neo* probe	ESC or mice
	*neo* or *lacZ* count by qPCR + vector backbone PCR	Mice

### High-Throughput Genotyping and QC Tests used by the Sanger Institute MGP

**Fig. 2:**
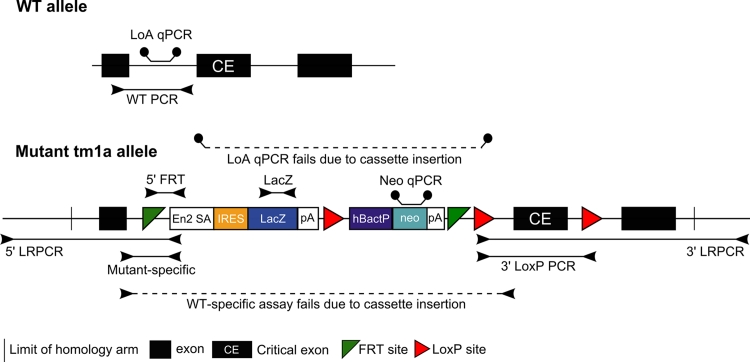
QC tests performed on mouse tissue samples (promoter-driven design shown for illustrative purposes). WT PCR: A gene-specific assay that detects only the wild-type allele. Insertion of the cassette makes the product too large to be amplified with the conditions used. Mutant PCR: A gene-specific assay that uses one gene-specific primer and one universal cassette primer and amplifies only the mutant allele. This can be used in conjunction with the WT PCR to genotype mice using gel-based methods. 5′FRT: Universal PCR assay to determine presence of the 5′ end of the cassette and 5′ FRT site. *neo* qPCR: Universal real-time PCR assay to determine the presence and copy number of the *neomycin* selection cassette. LacZ: a universal PCR assay to determine presence/absence of the *lacZ* gene. LoA qPCR: Loss of WT allele qPCR assay that determines the copy number of the WT (nontargeted) allele. Targeted clones will see a reduction in copy number. LoxP: a universal assay to determine presence of the *loxP* site 3′ to the critical exon. Gene-specific primers can also be used if the critical exon region is very large. LRPCR: Long-range PCR pairs one primer within the cassette with a gene-specific primer outside of the homology arm and is used to confirm the targeting of the allele. Two PCR-based tests are also used to detect the presence of vector backbone incorporation into the genome, which would suggest an improper targeting event

For rapid and universal detection of potential germline transmission of the mutant allele from the initial breeding of chimeras (crossed to C57BL/6N Taconic), G_1_ carriers are identified by a universal qPCR assay designed to the *neomycin* selection marker in the targeting cassette. Gene identification and QC of the allele are then performed on all G_1_ heterozygotes before switching to the *neo* qPCR for routine genotyping and phenotyping cohort production. Gene-specific assays and further QC are then performed on selected homozygous mutant animals before and after phenotyping. The QC methods performed on mice are outlined in Fig. 2. They are a mixture of end-point short range PCR (srPCR) and copy number counting qPCR-based assays designed to both the mutant and wild-type (WT) alleles. The presence or absence of the 3′ *loxP* site in conditional ready “knockout-first” lines was determined by either a universal PCR assay (primers designed to the cassette and linker sequences 3′ to the *loxP* insertion site) or, where no product was detected, a PCR using two gene-specific PCRs followed by sequencing.

Further details of the tests used at the Sanger Institute, including primer sequences and reaction conditions, can be found in Supplementary Information S1 and also in the IKMC knowledge base (http://www.knockoutmouse.org/kb/2).

## Results

During the period between September 2006 and November 2011, a total of 731 EUCOMM/KOMP ESC clones were microinjected (582 MGP, 94 EUMODIC, and 48 KOMP2-funded) and subsequently achieved germline transmission, of which 632 mouse colonies (86 %) passed QC.

**Fig. 3:**
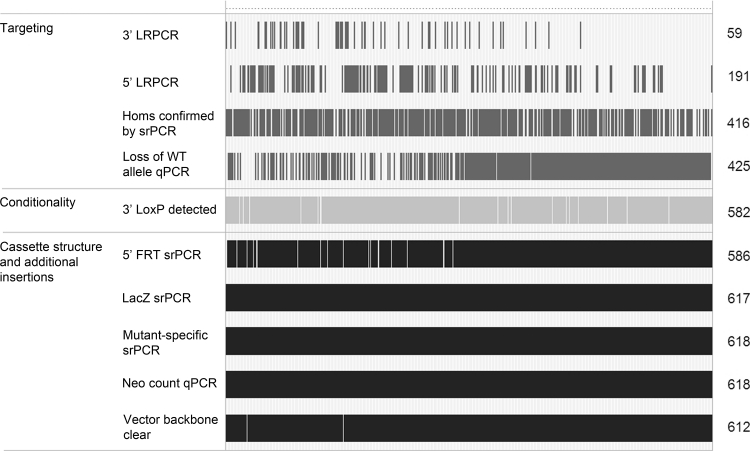
Quality control status for mouse strains made available to the community. Each stroke represents one test performed per mouse colony. The majority of targeting confirmation is provided by loss of WT allele qPCR and/or loss of a wild-type amplicon using gel-based short-range PCR. Strains that have lost the 3′ *loxP* and therefore the conditionality capability are still made available to the research community as they may be of use as a loss-of-function mutant

Details of the assays that have been performed on the released lines are shown in Fig. 3; not all assays were completed on all lines released to the community as the QC methods evolved as the MGP has progressed and gained experience with the KOMP and EUCOMM ES cell resource.

### Analysis of Lines that Failed QC

#### Correct Gene Targeting (Gene id and Mutation Structure)

A total of 99 lines did not pass our QC protocol, 14 % of the total transmitting clones, the reasons for which are summarized in Table 2. Failures in the experimental protocols can be categorized into two main classes: technical problems and real gene-targeting errors. As a general workflow, when a QC failure was obtained it was assumed to be a technical problem and further effort was made to verify the correct gene targeting. A small subset of lines that failed targeting by PCR methods were analysed by Southern blot and also showed incorrect targeting (data not shown). Lines that initially failed were investigated with additional tests to confirm the results, usually by analysis of a qPCR assay designed to the critical exon (CE) region (if more than two copies are detected in heterozygotes or homozygotes compared to wild types, it confirms that the targeting is incorrect) or by redesign of PCR primers. A subset of lines subsequently passed QC on further testing and are not included in Table 2; *Zfp106* EPD0033_4_C03, for example, failed to confirm homozygotes detected by qPCR with a srPCR assay, but correct targeting was suggested by LRPCR and gene expression analysis. The mutant allele design for *Zfp106* is in an area of high-density repeats which resulted in the initial srPCR assays amplifying nonspecific products; subsequent assay redesign produced the desired result which was later confirmed by loss of WT allele qPCR. The colony for *Kng2* EPD0554_8_A01 failed loss-of-WT-allele qPCR at the G_1_ stage but passed both 5′ and 3′ LRPCR. Analysis of the cassette insertion region of the mutant allele revealed that 408 bp of the flanking sequence was duplicated in *Kng1* and prevented the qPCR assay from accurately detecting the loss of copy number. The WT and mutant PCR assays were redesigned to avoid the duplicated region and the targeting confirmed by failure to amplify a WT band in homozygotes detected by *neo* count qPCR.

**Table 2:** QC failures mouse colonies

Reason for QC failure	No. of lines	% total lines
Incorrect targeting	58	7.9
5′ end of cassette missing and incorrect targeting	24	3.3
Incorrect *neo* count	11	1.5
Incorrect *neo* count and incorrect targeting	4	0.5
5′ end of cassette missing	2	0.3
Total	99	13.5

Most cases of QC failures involving the cassette structure were due to a deletion of the 5′ end. To investigate whether the size of the deletion was variable or from a fixed point, a tiling PCR assay covering the length of the L1L2_Bact_P cassette (the most frequently used in the EUCOMM/KOMP-CSD resource) was designed and tested. We found that the amount of genetic material deleted was not constant between the QC-failed lines tested; for example, the *2210012G02Rik* EPD0131_3_F05 line showed a deletion of the splice acceptor and most of the IRES element (Supplementary Information S2), whereas *Myo10* EPD0272_4_C10 had a deletion of the entire cassette up to the *neo* selection marker. Some issues were also observed internal to the cassette; *Btbd11* EPD0463_1_A11 was shown to carry a deletion of 929 bp located 940 bp 3′ of the *lacZ* gene initiation site. The original “final vector” DNA and four alternative ESC clones were checked and did not carry this deletion, suggesting that it occurred during electroporation and subsequent homologous recombination (Supplementary Information S3).

We found that the cell line used also had a significant effect on the subsequent QC status. Colonies from the JM8.F6 cell line showed 50 % fewer QC failures (12/159, *P* = 0.0216), whereas those from the JM8A1.N3 cell line produced over twice as many failures than the average failure rate (13/43, 30 %, *P* = 0.0182). This suggests that the JM8A1.N3 cells may have a greater proportion of mixed populations of targeted and nontargeted clones compared to the other lines, and the nontargeted cells then go on to constitute the germ cells of the chimeras. The JM8A3.N1 and JM8.N4 cell lines did not show a significant difference (*P* = 0.202 and *P* = 0.582, respectively).

No significant difference (*P* = 0.123) was detected with the type of mutation used for the allele from conditional-ready designs (91/608 lines) compared to deletion-based designs (8/27 lines).

### Loss of the 3′ loxP Site

The 3′ *loxP* site can be lost from the mutant allele during homologous recombination as it is embedded within the 3′ homology arm and at a distance from the selection cassette. From a total of 600 of “knockout-first conditional ready” tm1a lines tested, we found 18 (3 %) that did not carry the 3′ *loxP* site that was detected during the original ES cell production screen, possibly due to mixed ESC colonies. As expected, this event is ESC clone-specific, and, thus, differences in presence/absence of the 3′ *loxP* site in different clones from the same electroporation for the same gene were observed in some cases where duplicate microinjections were performed. For example, the genes *Mlec*, *Smyd5*, and *Pabpc4*: mice derived from clones EPD0600_1_A06, EPD0027_5_G01, and EPD0025_3_C07 did not possess a 3′ *loxP* site, whereas those derived from EPD0600_1_H03, EPD0027_5_A02, and EPD0025_3_C08 did. These results highlight the need to reconfirm the presence of the 3′ *loxP* site in the mice generated from the ESC resource if conditional mutants are needed in a downstream research. Lines that do not possess the 3′ *loxP* site are still useful and are made available to the scientific community by Sanger MGP, but as tm1e “targeted nonconditional” mutants.

### Evidence for Mixed ESC Populations

The discordance between the targeting screens performed at ESC clone production and the subsequent failure rate in mouse colonies may be due to (1) a mixture of targeted and nontargeted clones in the ESC population (where the nontargeted cell contamination preferentially contributes to the germline in the chimera), (2) a higher than expected false-positive PCR rate in the ESC screening during production, or (3) incomplete assessment of the ES cells resulting in structural and targeting issues being missed even if the cell population was pure.

An additional long-range PCR QC step on the ES cells based on either the 5′ or the 3′ homology arm of the mutant allele prior to microinjection did not reduce the subsequent failure rate in mouse colonies. This was unexpected and suggests that mixed-cell populations are a major factor; the end-point-based LRPCR reaction detects the targeted cells but does not give information that nontargeted cells are also present.

Further evidence for mixed populations of ESC colonies was detected in a small number of mouse colonies, where transmission of two different alleles was detected by analysis of the G_1_ (chimera × C57BL/6N Taconic) animals. In most cases, these originated from the different chimeras [examples include *Tmem126a* EPD0409_3_A09 (Supplementary Information S4), *Slc25a21* EPD0085_1_D04, *G3bp2* EPD0598_4_D01, *Ide* EPD0158_4_G09, and *Mtap2* EPD0416_2_A02]. These incorrect alleles were not selected for further expansion. Multiple-targeting events were also observed originating from the same chimera [e.g., *Srrm4* EPD0538_3_A07 (Supplementary Information S4), *Bai1* EPD0675_3_C01, and *Rftn2* EPD0176_4_A01], where some offspring showed correct targeting and cassette structure and other heterozygous littermates did not.

Evidence for incorrect targeting events of the mutant allele is exemplified by *Tcf7l2* EPD0130_2_C06 and *Crtc2* EPD0197_3_C08; both passed the 5′ and 3′ LRPCR QC assays in the mouse line but failed to detect a loss in copy number of the WT allele by qPCR. An additional copy of the floxed CE region was also detected, suggesting that the mutant allele had targeted the correct locus but not completely replaced the endogenous form.

These results underline the need to carefully check each G_1_ individual used for expanding the colony, as transmission of the incorrect allele may seriously affect the utility of the mouse line or give misleading phenotyping results. With a few additional QC steps, however, any issues discovered at this early stage can easily be filtered out and the correctly targeted mice then used to expand the colony. Although these mixed events were a small percentage (~2 %) of the overall numbers of lines produced, they can result in a disproportionate amount of effort and costs needed to correct them once the colony has expanded, if they are detected at all.

However, one incorrect clone does not mean all clones for that gene are incorrect; in some cases where lines had failed QC, alternative clones were microinjected and subsequently passed. For example, the gene *Trim66*: mice derived from ESC clone EPD0027_3_D06 failed targeting QC (LoA qPCR failed, homozygotes by qPCR not confirmed by srPCR), whereas mice derived from clone EPD0155_5_A11 using an alternative design passed (LoA qPCR passed, homozygotes by qPCR confirmed by srPCR). Another example is the gene *Twf1*; mice derived from ESC clone EPD0127_5_C07 failed targeting (homozygotes by qPCR not confirmed by srPCR) and *neo* qPCR QC, but the line derived from EPD0127_5_E05 passed (5′ and 3′ LRPCR amplification, homozygotes by qPCR confirmed by srPCR). These experiments help validate the resource as a whole and show that even if one clone may be incorrect, others in the collection for that gene may be correctly targeted.

## Discussion

With all high-throughput projects there is an expected degree of trade-off between the accuracy of the resource and the rate of generation (Gerhard et al. 2004; Ryder et al. 2004). The main method used for the EUCOMM/KOMP resource in screening the ES cell clones during production was by long-range PCR and sequencing, using one primer in the cassette and one beyond the limit of the homology arms of the construct design (most frequently at the 3′ end). Although this method allows rapid detection of correct targeting, it cannot detect a mix of targeted and nontargeted clones, which would require a quantitative PCR approach or Southern blot analysis.

We found that the use of additional long-range PCR assays across the 5′ homology arm performed on ESC colonies did not provide any improvement in the transmission of correctly targeted events, which suggests that mixed ESC clones may be the cause of most of the targeting issues observed. To estimate the frequency of potentially mixed clones, we selected the subset of clones that passed additional LRPCR QC (by either the 5′ or the 3′ end) prior to microinjection and calculated how many then failed QC at the mouse stage (Supplementary Information S5). This method, of course, would not detect mixed clones which then contributed the correct cells to the mouse embryo, so this calculation may be an underrepresentation of the true value.

The reason for mixed-cell populations is most likely the practical limitations of the very-high-throughput nature of the ESC generation of the EUCOMM/KOMP-CSD project, where colonies are manually picked from culture plates; e.g., the JM8A1.N3 cells were much harder to culture and process in the laboratory, which may account for the higher percentage of mixed clones compared to the other lines. However, the contribution of this particular cell line to the total number of targeted alleles in the EUCOMM/KOMP-CSD collection is less than 15 %, compared to 60 % from the JM8A3.N1. More quantitative, yet practical, pre-microinjection QC methods such as loss-of-allele assays (Valenzuela et al. 2003) are required to reduce the transmission of incorrect alleles. QC failure does not represent a problem for the resource since in a great majority of cases there are alternative clones that can be injected for each allele. If alternative clones are not available, however, mixed clones may be rescued by subcloning. When the presence of the 3′ *loxP* site in “conditional ready” mutants in the collection was analysed, 97 % of strains’ genes tested displayed the expected results. The small number of conflicts with the *loxP* results could be due to a mixed colony of conditional and nonconditional targeted clones or a low rate of false-positive PCRs during the screening.

Our results highlight the importance of confirming the structure of the targeted mutation in strains derived from the EUCOMM/KOMP-CSD resource. Ideally, this can be achieved with Southern blot analysis of the targeted mutation using external probes. In a high-throughput environment we have replaced this technique with a suite of PCR and qPCR assays that yield the same level of QC. All QC assay results performed on mouse lines are displayed on the IKMC (www.knockoutmouse.org) and EMMA (www.emmanet.org) websites. It is important to note that genotyping mice purely by short-range PCR without reconfirming the targeting is risky; nontargeted lines may appear to be homozygous-lethal, as the WT-specific assay will always amplify a product.

In order to continue to unify the mouse QC for IKMC partners and the newly established IMPC (Brown and Moore 2012), and further simplify the interpretation of results for researchers, we propose here a confidence scoring system for the QC categories based on a four-character code. This is summarized in Table 3; scores are assigned based on the level achieved, ranging from no additional QC to whole-genome sequencing. For example, the line *Zfp106* EPD0033_4_C03 would be ***7CCC*** and *Nek10 EPD0135_5_C07* would be ***5CCC***. Under this system, the majority of Sanger MGP lines would be ***5CCC***, with over 95 % of the collection having a targeting score of 5 or over. This method can also be extended for ESC QC and incorporate additional categories as required (e.g., karyotyping of cells by either chromosome spreads or qPCR-based assays prior to microinjection).

**Table 3:** Proposal for a serial code for rapid and comprehensive display of mouse QC

Index	Targeting	Index	3’ *loxP*	Index	Cassette structure:	Index	Additional insertions:
1	No confirmation beyond ESC screen/QC	A	No verification beyond ESC screen/QC	A	No verification beyond ESC screen/QC	A	No verification beyond ESC screen/QC
2	Either 5′ or 3′ LRPCR amplification of a band	B	Amplification using qPCR-based universal assay	B	srPCR based assays at various points along cassette (e.g., *lacZ*, *neo*, 5′ FRT)	B	Vector backbone PCR
3	Both 5′ and 3′ LRPCR amplification of a band	C	Amplification using srPCR-based universal assay	C	qPCR based assays at various points along cassette (e.g., *lacZ*, *neo*, 5′ FRT); exclusive or in combination with step B	C	*neo* or *lacZ* count qPCR plus step B
4	Step 3 plus end sequence confirmation	D	Amplification using gene-specific srPCR-assay	D	Amplification of PCR tiling array across whole cassette	D	Southern blot
5	Loss of WT allele qPCR and/or srPCR confirmation of homozygotes	E	Sequencing of PCR product from C or D	E	Southern blot	E	Genome sequencing of mouse
6	Southern blot or steps 3 and 5	Z	No *loxP* in design or no *loxP* detected	F	Full sequencing of cassette		
7	Steps 3 and 5 (or step 6), and gene expression analysis showing knockout/down of targeted allele						
8	Genome sequencing of mouse						

The EUCOMM/KOMP-CSD mutant ES cell collection is an extremely valuable resource for the scientific community. Our data suggest that, in the absence of any additional pre-microinjection QC, 86 % of the ESC clones that achieve GLT produce strains with correctly targeted events, and that a few simple QC assays at the G_1_ chimera progeny stage can rapidly screen out the majority of incorrect events (for scientists ordering ESC clones from repositories, requesting three clones should give a 99.7 % chance that at least one is correctly targeted). This will not only save money and effort, it will also help reduce the number of experimental animals used, in compliance with the 3Rs (Fenwick et al. 2011; National Centre for the Replacement, Refinement and Reduction of Animals in Research (NC3Rs) Mission and Strategy 2012).

## Electronic supplementary material

Below is the link to the electronic supplementary material.

Supplementary material (PDF 686 kb)

Supplementary material (XLSX 90 KB)
