# Supplementary material for: Molecular Characterization of Mutant Mouse Strains Generated from the EUCOMM/KOMP-CSD ES Cell Resource
Source: Mamm Genome. 2013 Aug 4;24(7):286–94. doi: 10.1007/s00335-013-9467-x (PMC3745610; doi:10.1007/s00335-013-9467-x)
Supplement: Supplementary file 1 — Supplementary material (PDF 686 kb) [file 335_2013_9467_MOESM1_ESM.pdf]

Supplementary information

S1 – Mouse QC tests

Short Range PCR reagents and cycling conditions

Reagent	volume (ul)
Gene specific Primer 1 (10uM)	0.3
Gene specific Primer 2 (10uM)	0.3
MgCl2 (50mM)	0.45
10x Buffer	1.5
dNTP (100mM)	0.15
Taq*	0.15
ddH$_2$O	11.15
DNA	1
**Total**	**15**

*Platinum Taq is used in the PCR reaction (Invitrogen)

PCR conditions (program name TA58)

1 94 ^{o}C 5 min
2 94 ^{o}C 30 sec
3 58 ^{o}C 30 sec
4 72 ^{o}C 45 sec
5 Go to ‘2’ + 34 cycles
6 72 ^{o}C 5 min
7 12 ^{o}C forever

qPCR reagents and cycling conditions

Reagent	Volume (ul)
2x GTXpress buffer*	5
60x TaqMan assay	0.166
ddH$_2$O	3.334
20x endogenous probe (Tfrc)*	0.5
DNA	1

*Applied Biosystems

95ºC 20sec } x1

95ºC 10sec
60ºC 30sec } x35

Loss of WT allele qPCR

The wild type loss of allele (WT LoA) qPCR assay uses a hydrolysis probe assay to determine the copy number of the wild type allele in a sample. Homozygote mutants will show no amplification, heterozygotes one copy and wild type mice will show two copies when compared to a WT control. The assay is usually designed in the region of the genome deleted during the mutant allele design phase (between the U5 and U3 sequences) although some sequence flanking the cassette insertion point can be used if necessary.

Assays are usually designed to amplify a product of less than 100bp as this helps to attain maximum efficiency of the PCR reaction, which is important for the downstream analysis and subsequent copy number determination (Sanger uses the 2-ddCt method for analysis). The WT LoA qPCR assay is useful for detecting that the correct locus has been targeted - a mis-targeting or incorrect allele replacement event will not disrupt the assay binding site and mutants will therefore show no loss in copy number.

Neo count qPCR

The neo count qPCR assay uses a hydrolysis probe assay to determine the copy number of the Neo cassette in a sample. Homozygote mutants will show two copies of neo, heterozygotes one copy and wild type mice will show no amplification when compared to homozygote controls.

The neo count does not confirm the correct targeting of the allele, nor the gene identity. It can however be used as a QC tool to detect multiple insertion events or concatemers within the cassette.

qPCR assays

Assay Name	F name	F sequence	R name	R sequence	Probe name	Dye	Probe sequence
LoxP	3LOXP_F	GAGATGGCGCAACGCAATTAA	3LOXP_R	GGCGAGCTCAGACCATAACTT	3LOXP_M	FAM	AATGTATGCTATACGAAGTTATC
LacZ	LacZ_F	GGAGTGCGATCTTCCTGAGG	LacZ_R	CGCATCGTAACCGTGCATC	LacZ_M	FAM	CGATACTGTCGTCGTCCCCTCAAACTG
Neo	Neo_F	GGTGGAGAGGCTATTCGGC	Neo_R	GAACACGGCGGCATCAG	Neo_M	FAM	TGGGCACAACAGACAATCGGCTG
5_FRT	5FRT_QPCR_F	CGATACCACGATATCAACAAGTTTG	5FRT_QPCR_R	GGGAAAGGGTTCGAAGTTCCT	5FRT_QPCR_M	FAM	CGCCGGAACCGAA

Short range PCR assays

Assay	Forward primer	F sequence	Reverse primer	R sequence	Size (bp)	PCR
5' FRT	5FRT_F	AGGCGCATAACGATACCACGAT	5FRT_R	CCACAACGGGTTCTTCTGTT	204	TA58
LacZ	LacZ_2_small_F	ATCACGACGCGCTGTATC	LacZ_2_small_R	ACATCGGGCAAATAATATCG	108	TA58
LoxP	Floxed PNF	ATCCGGGGGTACCGCGTCGAG	Floxed LR	ACTGATGGCGAGCTCAGACC	variable	TA58

Universal cassette primer used in mutant-specific PCR assay

Primer name	Sequence
CAS_R1_Term	TCGTGGTATCGTTATGCGCC

Targeting Vector Backbone Assay

Primers are designed to amplify sequences present only in the vector backbone. If a correct homologous recombination has occurred then no product should be amplified. If a product is detected it strongly suggests an incorrect targeting event or a multiple insertion event.
Not all vector primers will be present in all backbones – please refer to the tables below on which primers to use.
Please note that the absence of a product does not necessarily prove that the targeting is correct and this test should be used in conjunction with others to confirm the targeting.

Primer	Sequence
VF1_F	CTCCTTTCGCTTTCTTCCCT
VF1_R	GCGAAAAACCGTCTATCAGG
VF2_F	TTGATTTGGGTGATGGTTCA
VF2_R	GAAATCGGCAACATCCCTTA
VF4_F	GTAGCTGACATTCATCCGGG
VF4_R	CTTTTCTACGGGGTCTGACG
VF5_F	CGAACGACCTACACCGAACT
VF5_R	AAAGATACCAGGCGTTTCCC
VF6_F	CAGCATGCCTGCTATTGTCT
VF6_R	TAGTTGCCAGCCATCTGTTG
VF7_F	GTGGCGCTTTCTCATAGCTC
VF7_R	GCCTACATACCTCGCTCTGC
VF8_F	ATAATACCGCGCCACATAGC
VF8_R	TTTGCCTTCCTGTTTTTGCT

Vector backbone primer combinations and different backbone types

Vector	VF1_F	VF1_R	VF2_F	VF2_R	VF4_F	VF4_R	VF5_F	VF5_R	VF6_F	VF6_R	VF7_F	VF7_R	VF8_F	VF8_R
L3L4_pZero_DTA_kan	y	y	n	n	y	y	y	y	n	n	n	n	n	n
L3L4_pZero_kan	y	y	y	y	y	y	y	y	n	n	n	n	n	n
L3L4_pD223_DTA_spec	n	n	n	n	n	y	y	y	y	y	n	n	n	n
L3L4_pD223_DTA_T_spec	n	n	n	n	n	y	y	y	y	y	n	n	n	n
R3R4_pBR_DTA+_Bsd_amp	n	n	n	n	n	n	n	n	n	n	y	y	y	y

LoxP Confirmation

Confirmation of the loxP site downstream of the critical exon can be achieved by several methods.
The primary method uses two universal primers to amplify across the critical exon region (Skarnes et al., 2011). Presence of a band (when accompanied by the proper controls) can be used as confirmation of the loxP site.
In some cases the region to be amplified may be too large for the standard PCR reaction, in which case a gene-specific primer can be used in conjunction with one of the universal primers or two gene specific primers can be used. The latter method will produce two bands of different sizes in heterozygotes, although this may be difficult to resolve on some gel systems.

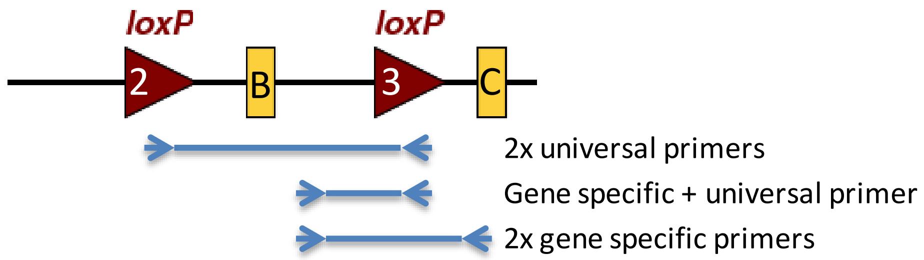

Mutant Specific short range PCR

The mutant-specific srPCR amplifies a product in the mutant form of the allele. This can be used in conjunction with a wild-type specific assay to determine the genotype of the mouse. Homozygous mice will produce no amplification in the wild type assay shown below (detection of homozygotes by this method can also be used to confirm the targeting).
The mutant specific PCR on its own is not sufficient to confirm the correct targeting as the primer sites are within the homology arms of the construct. It can be used however to check the gene identity of the injected construct.

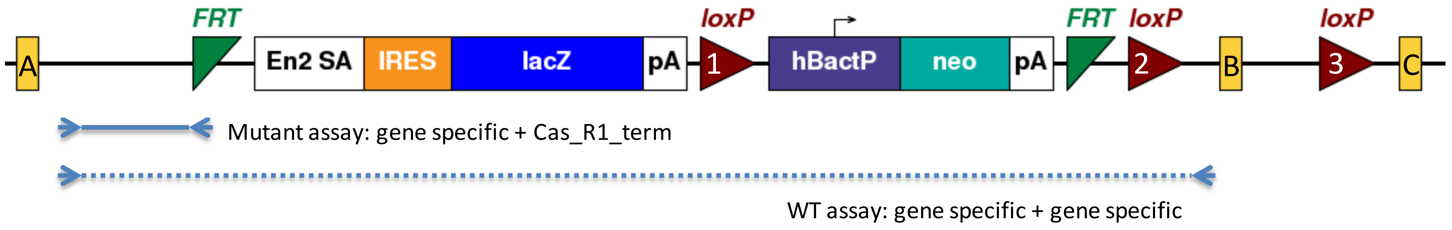

S2. Cassette deletion in *2210012G02Rik* shown by a PCR tile. *Spns2* is shown as a positive control

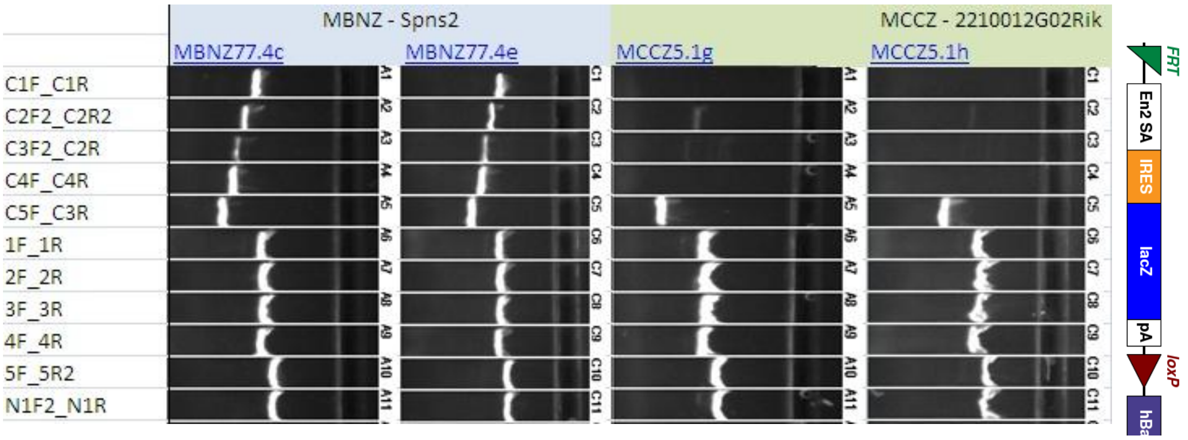

A PCR tiling path was designed across the L1L2_Bact_P cassette (Skarnes et al., 2011); deleted portions of the cassette are apparent by the lack of amplification of a band on a gel - in this example the *2210012G02Rik* is missing the *En2* splice acceptor and the IRES element. The structure of the cassette is shown on the right hand side

S3. Internal deletion of the *lacZ* cassette detected in *Btbd11* EPD0463_1_A11.

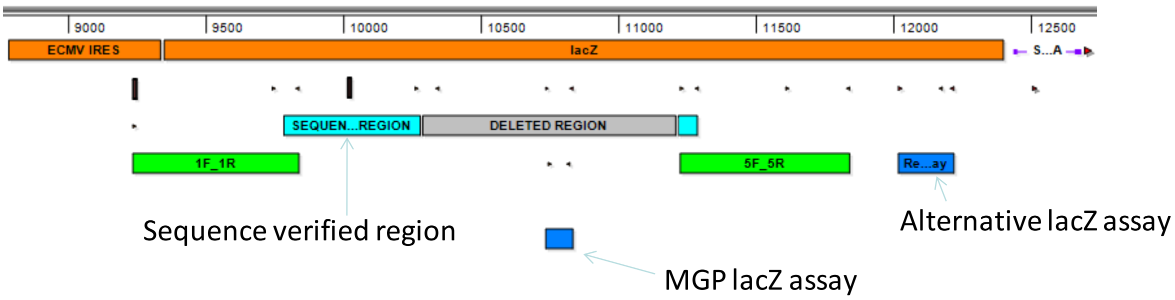

The internal cassette deletion was originally detected by a lack of amplification in the MGP *lacZ* assay and then confirmed by tiling-PCR and sequencing across the affected region. An alternative assay can be used for genotyping these mice if required.

The structure of the cassette is shown as orange bars at the top with the sequence-verified region of the *lacZ* in blue, and the confirmed deletion in grey. 1F_1R and 5F_5R represent amplicons flanking the deleted region.

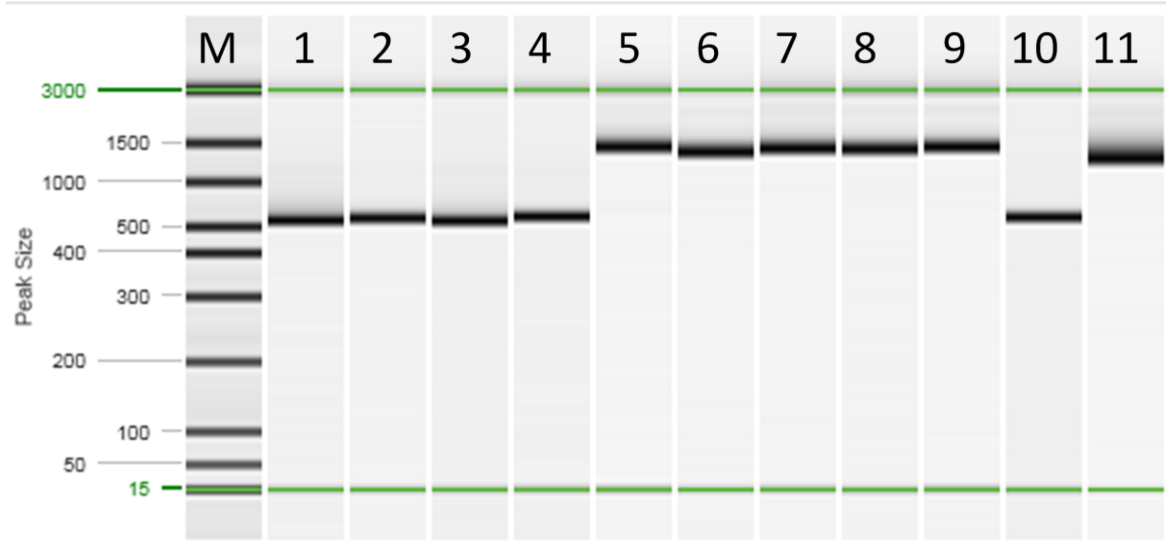

Gel image showing confirmation of the *lacZ* deletion using primers flanking the deleted area.

Lanes 1-4: MDAR *Btbd11*$^{tm1a(EUCOMM)Wtsi}$ mice possessing the *lacZ* deletion amplify a 630bp product

Lane 5: Positive control mouse

Lanes 6-10: Different ESC clones from Btbd11 electroporation EPD0463. Only the clone EPD0463_1_A11 (lane 10) shows the deletion

Lane 11: Final vector PGRS0001_B_H11 DNA does not show a deletion and amplifies an expected product of 1.55kb

Lane M: size marker. Lane 7. Negative control.

The upper and lower green bands are alignment markers used by the fragment analysis software (Qiagen)

S4. QC issues detected in mouse colonies

Tmem126a targeting confirmation.

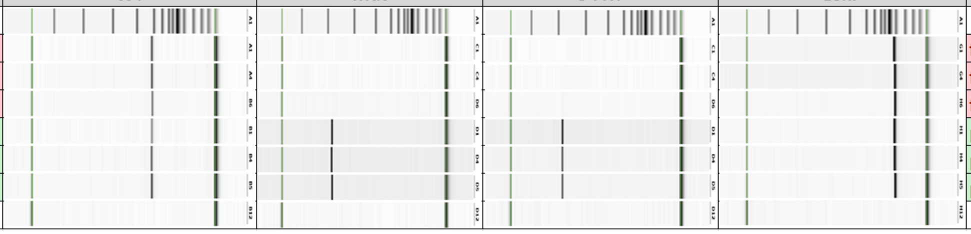

Mouse ID	WT	Mut	5'FRT	LoxP	Loss of WT allele qPCR
MEJS9.1a					fail
MEJS9.1d					fail
MEJS10.2a					fail
MEJS7.1a					pass
MEJS8.1a					pass
MEJS8.1b					pass
-ve control					

Tmem126a shows different targeting events from the two founding chimeras. Results from four assays performed by srPCR (wildtype, mutant, 5' cassette and 3' *loxP* and visualised on a Qiagen

QIAxcel) and from the loss of WT allele qPCR are shown. The upper and lower bands are alignment markers used by the fragment analysis software (Qiagen)

G_1 mice MEJS9.1a,9.1d and 10.2a (highlighted red) show no amplification of the 5' end of the cassette, no amplification of the mutant-specific PCR (indicating that a deletion of the 5' end of the cassette has occurred) and a targeting QC fail in the WT loss of allele qPCR. Mice MEJS7.1a, 8.1a and 8.1b (highlighted green) show correct cassette structure and correct targeting

Srrm4 targeting confirmation

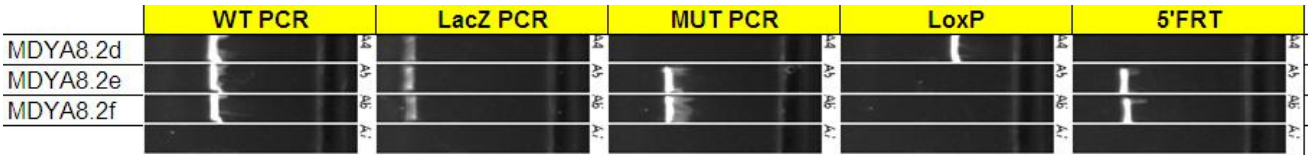

G_1 siblings containing a mixture of different gene targeting events in *Srrm4* (EPD0538_3_A07). MDYA 8.2e and 8.2f show the expected cassette structure (wildtype, lacZ, 5' cassette and mutant-specific assays all amplify a band) but the 3' *loxP* assay fails, indicating that this is a tm1e targeted non-conditional strain. MDYA8.2d shows a possible deletion of the 5' end of the cassette (mutant-specific and 5' cassette assays fail) but does amplify a *loxP* product.

S5. Mixed ESC populations per cell line

Based on ESC clones which passed an additional long range PCR step prior to micro-injection but then later failed QC as mouse strains.

Cell line	QC fail	QC pass	Total strains	% QC fail
JM8.F6	2	57	59	3.4%
JM8.N4	12	113	125	9.6%
JM8A1.N3	11	29	40	27.5%
JM8A3.N1	35	173	208	16.8%
**Total**	**60**	**372**	**432**	**13.9%**

References

Skarnes, W. C., Rosen, B., West, A. P., Koutsourakis, M., Bushell, W., Iyer, V., Mujica, A. O., et al. (2011). A conditional knockout resource for the genome-wide study of mouse gene function. *Nature*, *474*(7351), 337–42. doi:10.1038/nature10163
